# Malnutrition in Healthy Individuals Results in Increased Mixed Cytokine Profiles, Altered Neutrophil Subsets and Function

**DOI:** 10.1371/journal.pone.0157919

**Published:** 2016-08-22

**Authors:** Y. Takele, E. Adem, M. Getahun, F. Tajebe, A. Kiflie, A. Hailu, J. Raynes, B. Mengesha, T. A. Ayele, Z. Shkedy, M. Lemma, E. Diro, F. Toulza, M. Modolell, M. Munder, I. Müller, P. Kropf

**Affiliations:** 1 Department of Medicine, Imperial College London, London, United Kingdom; 2 Leishmaniasis Research and Treatment Centre, Gondar University, Gondar, Ethiopia; 3 Department of Immunology, University of Gondar, Gondar, Ethiopia; 4 Department of Microbiology, Immunology and Parasitology, Addis Ababa University, Addis Ababa, Ethiopia; 5 Department of Immunology and Infection, London School of Hygiene and Tropical Medicine, London, United Kingdom; 6 Department of Epidemiology and Biostatistics, University of Gondar, Gondar, Ethiopia; 7 Department of Mathematics and Statistics, University of Hasselt, Hasselt, Belgium; 8 Department of Internal Medicine, University of Gondar, Gondar, Ethiopia; 9 Department of Cellular Immunology, Max-Planck-Institute for Immunobiology and Epigenetics, Freiburg, Germany; 10 Third Department of Medicine (Hematology, Oncology, and Pneumology), University Medical Centre Mainz, Mainz, Germany; Dasman Diabetes Institute, KUWAIT

## Abstract

Malnutrition is commonly associated with increased infectious disease susceptibility and severity. Whereas malnutrition might enhance the incidence of disease as well as its severity, active infection can in turn exacerbate malnutrition. Therefore, in a malnourished individual suffering from a severe infection, it is not possible to determine the contribution of the pre-existing malnutrition and/or the infection itself to increased disease severity. In the current study we focussed on two groups of malnourished, but otherwise healthy individuals: moderately malnourished (BMI: 18.4–16.5) and severely malnourished (BMI <16.5) and compared several immune parameters with those of individuals with a normal BMI (≥18.5). Our results show a similar haematological profile in all three groups, as well as a similar ratio of CD4^+^ and CD8^+^ T cells. We found significant correlations between low BMI and increased levels of T helper (Th) 1 (Interferon (IFN)-γ, (interleukin (IL)-2, IL-12), Th2 (IL-4, IL-5, IL-13), as well as IL-10, IL-33 and tumor necrosis factor-α, but not IL-8 or C reactive protein. The activities of arginase, an enzyme associated with immunosuppression, were similar in plasma, peripheral blood mononuclear cells (PBMC) and neutrophils from all groups and no differences in the expression levels of CD3ζ, a marker of T cell activation, were observed in CD4^+^ and CD8^+^T cells. Furthermore, whereas the capacity of neutrophils from the malnourished groups to phagocytose particles was not impaired, their capacity to produce reactive oxygen species was impaired. Finally we evaluated the frequency of a subpopulation of low-density neutrophils and show that they are significantly increased in the malnourished individuals. These differences were more pronounced in the severely malnourished group. In summary, our results show that even in the absence of apparent infections, healthy malnourished individuals display dysfunctional immune responses that might contribute to increased susceptibility and severity to infectious diseases.

## Introduction

Undernutrition, here referred to as malnutrition, is a result of inadequate diet and/or malabsorption of nutrients. An estimated one out of ten people in the world are malnourished, of those 95.9% are living in the developing world [1]. Protein energy malnutrition (PEM) has been associated with different infectious diseases including malaria, tuberculosis, lower respiratory tract infections and diarrheal diseases; and these are the major cause of morbidity and mortality in developing countries [2, 3][4, 5]. Malnutrition is thought to be one of the major causes of immunodeficiency: in malnourished patients, both innate and acquired immunity are affected [6, 7]. Common immune defects are an imbalance in the ratio of CD4/CD8^+^ T cells [8], low expression levels of CD69 on lymphocytes [9], biased T helper cell responses [10, 11], reduced antibody responses [8]; impaired phagocytosis by macrophages [12, 13], lower nitrite/nitrate concentrations in wound fluid [14] and decreased NF-kappaB activation by macrophages [15] have also been shown in experimental models of PEM. Moreover, it has been shown that malnutrition and the subsequent impaired immune responses reduce the efficacy of oral vaccines in developing countries [16].

Despite the fact that it is generally accepted that malnutrition plays a crucial role in increased susceptibility to infection and/or disease severity by weakening the immune system, the causal links between malnutrition and infections are not yet well established. Since infection can also cause malnutrition through several factors such as decreased appetite and increased catabolism [17], it is difficult to identify the contribution of pre-existing malnutrition and/or infection-induced malnutrition to increased disease severity [18, 19]. Importantly, the majority of the work on malnutrition has been mainly done with malnourished patients suffering from infectious diseases, or other pathological conditions, and, apart from studies on patients with eating disorders such as anorexia nervosa [20, 21], little is known about the impact on malnutrition on the immune system of "apparently healthy" adult individuals.

Our previous work studying the immune responses of patients with visceral leishmaniasis (VL), a potentially fatal infectious disease caused by *Leishmania* parasites, has shown that the majority of these patients suffer from severe malnutrition [22, 23]. The immune status of these patients is characterised by a profound suppression of T cell responses, high levels of cytokine and chemokine production and strong inflammatory responses (reviewed in [24, 25]. These patients are living in the North West of Ethiopia, where malnutrition appears to be relatively common [26], however, precise information about adult malnutrition is scarce in Ethiopia.

Here, we performed a prospective study with "apparently healthy" individuals with normal and low BMI, living in the North West of Ethiopia. Our aim was to compare, in malnourished individuals and individuals with normal BMI, several immunological parameters that have been shown to be impaired in patients with nonhealing VL: haematological profile, cytokine profiles in the plasma, CD4^+^ and CD8^+^ T cell ratio and activation status, as well as neutrophil effector functions.

## Material and Methods

### Subjects and sample collection

The study was approved by the Institutional Review Board of the University of Gondar (IRB, reference SBMLS/1199/07). For this cross-sectional study, "apparently healthy" individuals were actively recruited from Gondar, Ethiopia and its surrounding areas; the aim of the project was explained to each individual in their native language, and during the period of our study (3 months), a cohort of 26 males and 17 females (Table 1) agreed to take part and signed a consent form. Before being included in the study, the participants were examined by a physician at the University of Gondar Leishmaniasis Research and Treatment Centre. In the present study, "apparently healthy " refers to individuals who had no signs of disease or physical condition that prevent them from engaging in physical activities and work; individuals who had no significant change in their BMI for the past 12 months, and who were reported as presenting with a low BMI (<18.5, referred to in this study as malnourished individuals) or a normal BMI (≥18.5, referred to in this study as normal BMI) by the physician, were included in the study. These inclusion criteria were based on clinical signs and symptoms and haematological tests (complete blood count), assessed by routine laboratory methods and physical evaluation. The cause for the low BMI of the cohort of malnourished individuals was described as lack of appetite and consequent low intake of food.

**Table 1:** Demographic data: age, sex and BMI.

Variables		Number	Percent (%)
Sex	Male	26	60.5
	Female	17	39.5
Age	<26 years	21	48.8
	26–30 Years	15	34.9
	>30 years	7	16.3
BMI	<16.5	14	32.6
	16-5-18.5	11	25.6
	>18.5	18	41.9

Nineteen individuals had a normal BMI (= ≥18.5, median BMI: 21.5 ±0.4) and twenty-six individuals had a low BMI (= <18.5, median BMI: 16.1±0.2). The malnourished group was further subdivided into two groups: moderately malnourished (BMI = 18.6–16.5, median BMI: 17.4±0.2) and severely malnourished (BMI = <16.5, median BMI: 15.5±0.2).

A total of 10ml blood was collected in heparinised blood tubes and the blood was processed within 10 minutes after blood collection: plasma was harvested on the top of the double density gradient performed to isolate PBMCs and neutrophils (NDGs) [27, 28] and was frozen immediately until further use. PBMCs and neutrophils were harvested from the two different layers, washed in phosphate buffered saline (PBS) and were divided into two parts: one was frozen immediately for the determination of arginase activity and one was used immediately for flow cytometry.

#### Haematological analysis

The haematological profiles were determined by using an automated CELL-DYN^®^ 1800 Haematology Analyser, USA.

#### Flow cytometry

*Characterization of T cells and neutrophils*: Antibodies used were as follows: for the neutrophil characterization, anti-CD15 (Clone H198, BD Pharmingen) labelled with Fluorescein Isothiocyanate (FITC), anti-arginase I (HyCult Biotechnology: clone 6G3) labelled with Allophycocyanin (APC) and anti-CD63 (BD Pharmingen: clone MOPC21) labelled with Phycoerythrin (PE) flurochromes. For T cell characterization, anti-CD4 (BD Biosciences: clone L200) labelled with FITC, anti-CD8 (BD Biosciences: clone RPA-T8) labelled with APC and anti-CD3ζ(Santa Cruz Biotechnology: clone 6B10.2) labelled with PE. Cells were washed with PBS, the fixation step was performed with 2% formaldehyde in PBS and the permeabilization step with 0.5% saponin in PBS. The percentages for the isotype controls were <1%.

*Phagocytosis*: *E*.*coli* particle suspension (pHrodo^®^ Red *E*. *coli* BioParticles^®^, Life Technologies) labelled with Alexa Fluor 488 was used to assess the phagocytic capacity of normal density granulocytes (NDGs). 1x10^4^ NDGs were incubated in RPMI containing 0.1% FBS with particles for 20min at 37°C as described in the manufacturer’s protocol, NDGs without beads were used as controls. The cells were then washed and analyzed by flow cytometry. To obtain the MFI shown in the figures, the MFI from NDGs incubated without beads was subtracted from the MFI from NDGs incubated with beads.

*Production of reactive oxygen species*: A kit (Total ROS detection kit, Enzo) was used to evaluate the production of reactive oxygen species (ROS) by neutrophils. 1x10^5^ NDGs were activated in RPMI containing 0.1% FBS with pyocyanin (a general ROS inducer) as described in the manufacturer’s protocol or with 1μg lipopolysaccharide (LPS, Sigma). NDGs without pyocyanin or LPS were used negative controls. The cells were analyzed by flow cytometry. To obtain the MFI shown in the figures, the MFI from NDGs incubated without activation was subtracted from the MFI from activated NDGs.

Acquisition was performed using a FACSCalibur (BD Biosciences) and data were analyzed using Summit v4.3 software.

#### ELISA

The ELISA for the determination of the acute phase C-reactive protein (CRP) was performed as described in [29]. IL-8 cytokine levels in plasma and supernatants were determined using a kit from eBioscience (Ready-SET-Go!ELISA Sets), according to the manufacturers’ protocol.

#### Luminex

Cytokine analysis for interleukin (IL)-2, -4, -5, -10, -12, -13, -33, TNF-α and IFN-γ in the plasma from individuals with low and normal BMI was performed by using the Luminex 200^™^ system (USA, Multiplex^®^ Map Kit) and the plate was analyzed using the Luminex 100 system. The readout for the concentration of each cytokine was detected as mean fluorescence intensity (MFI) by the instrument. These values were subsequently converted to pg/ml of cytokine based upon the MFI values from a set of standards that were run simultaneously in the assay.

#### Arginase activity

The enzymatic activity of arginase was measured as previously described [30] and to determine arginase activity in the plasma, urea concentrations were first determined in the plasma without the activation and hydrolysis steps; these values were subtracted from those obtained by measuring the urea.

One unit of enzyme activity is defined as the amount of enzyme that catalyzes the formation of 1 μmol of urea per min.

#### Statistical analysis

Data were evaluated for statistical differences using, Kruskal-Wallis, Spearman and Multivariate Analysis of Variance (MANOVA) tests (GraphPad Prism 6) and differences were considered statistically significant at *p*<0.05. Unless otherwise specified, results are expressed as median± SEM.

## Results

### Haematological profile

We first assessed the haematological profile of our cohort and as shown in Table 2, no significant differences (*p*>0.05) were observed in white blood cells (WBC), neutrophils, lymphocytes, red blood cell (RBC), haemoglobin (Hgb) and platelet counts between individuals with a normal BMI (>18.5), moderately malnourished (16.5–18.4) and severely malnourished (<16.5) individuals (Table 2). Since univariate analysis can be missleading, we analyzed the data presented in Table 1 using a MANOVA model in which the different variables of the haematological profiles are analyzed simultaneously. Our results indicate that there is no significant difference across the BMI groups (Table A in S1 Data).

**Table 2:** Haematological profile and percentages and ratio of CD4^+^ and CD8^+^ T cells of individuals with normal and low BMI.

BMI	WBC	Neutrophils	Lymphocytes	RBC	Hgb	Platelets	%CD4^+^ T cells	%CD8^+^ T cells	Ratio CD4/CD8
**≥18.5**	6.7±0.6	3.3±0.4	2.4±0.1	5.2±0.2	15.7±0.4	256.0±12.4	34.7±2.2	31.4±2.2	1.1±0.2
**16.5–18.4**	7.6±0.6	4.4±0.5	2.8±0.2	5.3±0.2	15.5±0.8	273.0±29.6	32.0±2.4	34.1±2.8	0.9±0.1
**<16.5**	6.1±0.6	3.2±0.3	2.6±0.2	5.1±0.2	15.4±0.5	282.0±13.1	36.0±2.5	27.7±2.2	1.1±0.2
***p* values Kruskal Wallis)**	0.5741	0.3567	0.7793	0.8174	0.9680	0.5775	0.3398	0.1276	0.4255

Blood was collected from individuals and the haematological profile (normal BMI: n = 17 and low BMI: n = 22) was determined using an automated CELL-DYN^®^ 1800 Haematology Analyser; the percentage and ratio of CD4^+^ and CD8^+^ T cells (normal BMI: n = 17 and low BMI: n = 21) were determined by flow cytometry. Statistical differences between the groups were determined using a Kruskal-Wallis test.

### Percentages and ratio of CD4^+^ and CD8^+^ T cells

It has been previously shown that the frequency of CD4^+^ and CD8^+^ T cells can be altered in malnourished individuals [31]. Here we measured the percentages and ratio of CD4^+^ and CD8^+^ T cells in PBMCs isolated from our cohort and our results show no significant differences in the percentages, and ratio of CD4 and CD8^+^ T cells in all groups (Kruskal-Wallis: Table 2 and Table B in S1 Data).

### Cytokines and inflammatory mediators

Cytokine production can be significantly altered as a result of malnutrition [32]. Here we assessed the levels of Th1, Th2, regulatory and other cytokines, as well as inflammatory mediators, in the plasma of our cohort.

**Fig 1:**
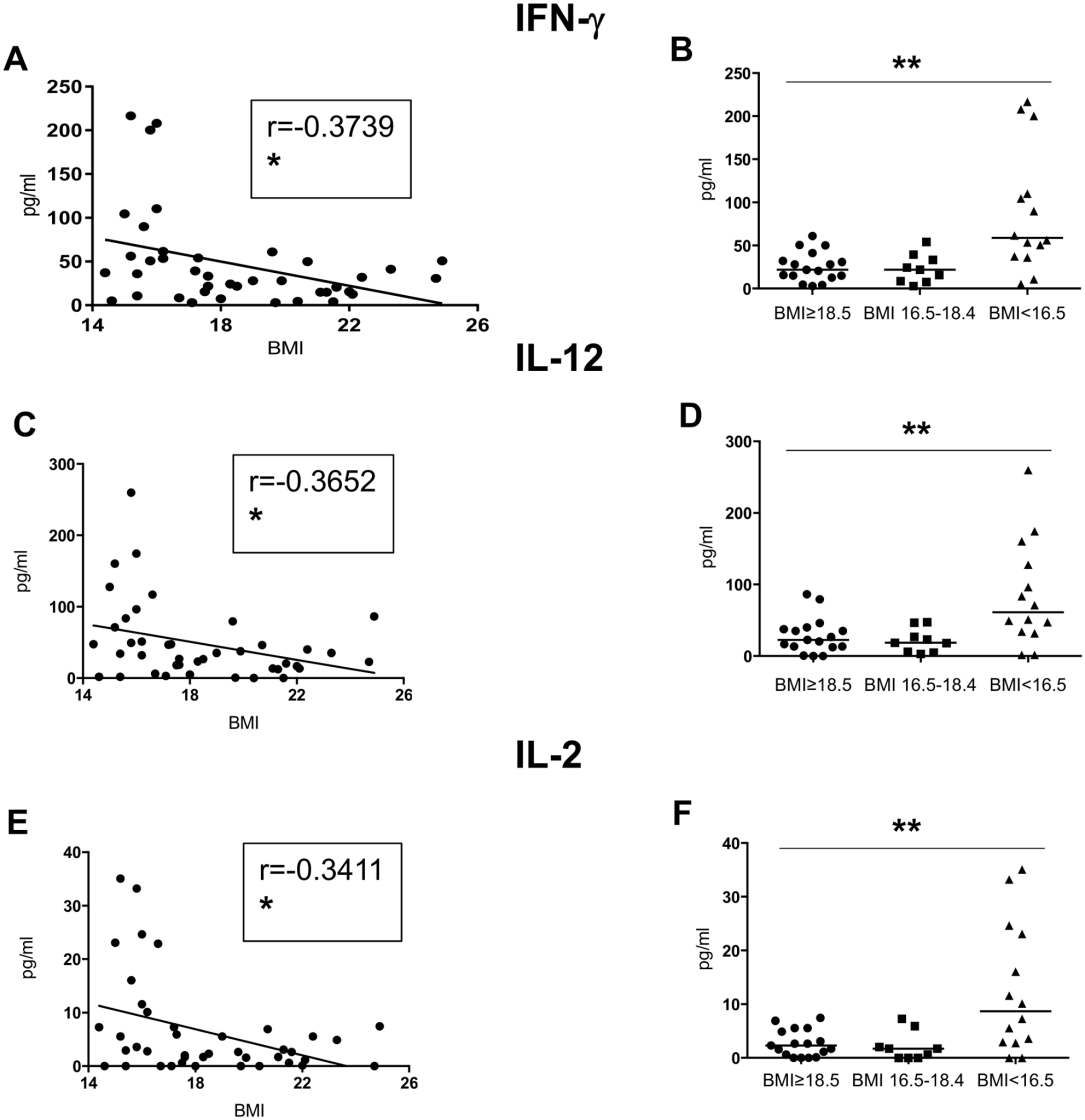
Levels of Th1 cytokines in plasma. The plasma from individuals with normal BMI (n = 17) and low BMI (n = 23, of which 9 were moderately malnourished and 14 were severely malnourished) was tested by Luminex for the levels of IFN-γ, IL-2 and IL-12. Fig A, C and E show correlations between BMI and the different cytokines and statistical significance was established using a Spearman rank test. Fig 1B, D and F show cytokine levels between the individuals with a normal BMI, moderately malnourished and severely malnourished individuals and statistical significance was established using a Kruskal-Wallis test.

Th1 cytokines (Table 3, Fig 1): Our results show significant negative correlations between the BMI and the plasma levels of IFN-γ, IL-12, and IL-2. Furthermore, significant differences were observed between the individuals with a normal BMI and those moderately and severely malnourished (Kruskal-Wallis and Spearman: Table 3 and Fig 1; Table C in S1 Data).

**Table 3:** Cytokines and inflammatory mediators in the plasma of individuals with normal and low BMI.

BMI	IFN-γ	IL-12	IL-2	IL-4	IL-5	IL-13	IL-10	IL-33	TNF-α	IL-8	CRP
**≥18.5**	21.7±4.2	22.6±6.0	2.3±0.6	0.13±0.03	3.9±0.7	23.7±8.3	2.8±0.7	0.0±16.3	11.8±3.9	3.8±0.6	9942±2760
**16.5–18.4**	21.7±5.6	18.7±5.5	1.7±0.9	0.10±0.03	2.5±0.3	26.7±5.8	2.6±4.3	0.0±12.2	19.0±3.0	1.5±0.5	3122±4053
**<16.5**	58.9±19.2	61.1±19.5	8.7±3.2	0.33±0.08	7.8±1.9	75.6±19.8	8.0±3.6	161.2±68.9	47.6±10.2	2.2±0.3	965±3807
***p* values (Kruskal Wallis)**	0.0024	0.0085	0.0085	0.0031	0.0080	0.0042	0.0186	0.0008	0.0029	0.2341	0.4799
**Correlation (BMI vs cytokines) *p* values (spearman)**	0.0175	0.0189	0.0291	0.0266	0.0309	0.0437	0.0284	0.0065	0.0387	0.4652	0.3060

The plasma from individuals with normal BMI (n = 17) and low BMI (n = 23, of which 9 were moderately malnourished and 14 were severely malnourished) was tested by Luminex (IFN-γ, IL-2, IL-12, IL-4, IL-5, IL13, IL-10, IL-33 and TNF-α) or by ELISA (IL-8 and CRP). Correlations between BMI and the different cytokine were assessed using a Spearman rank test. Statistical differences in cytokine levels between individuals with a normal BMI, moderately malnourished and severely malnourished individuals were established using a Kruskal-Wallis test.

**Fig 2:**
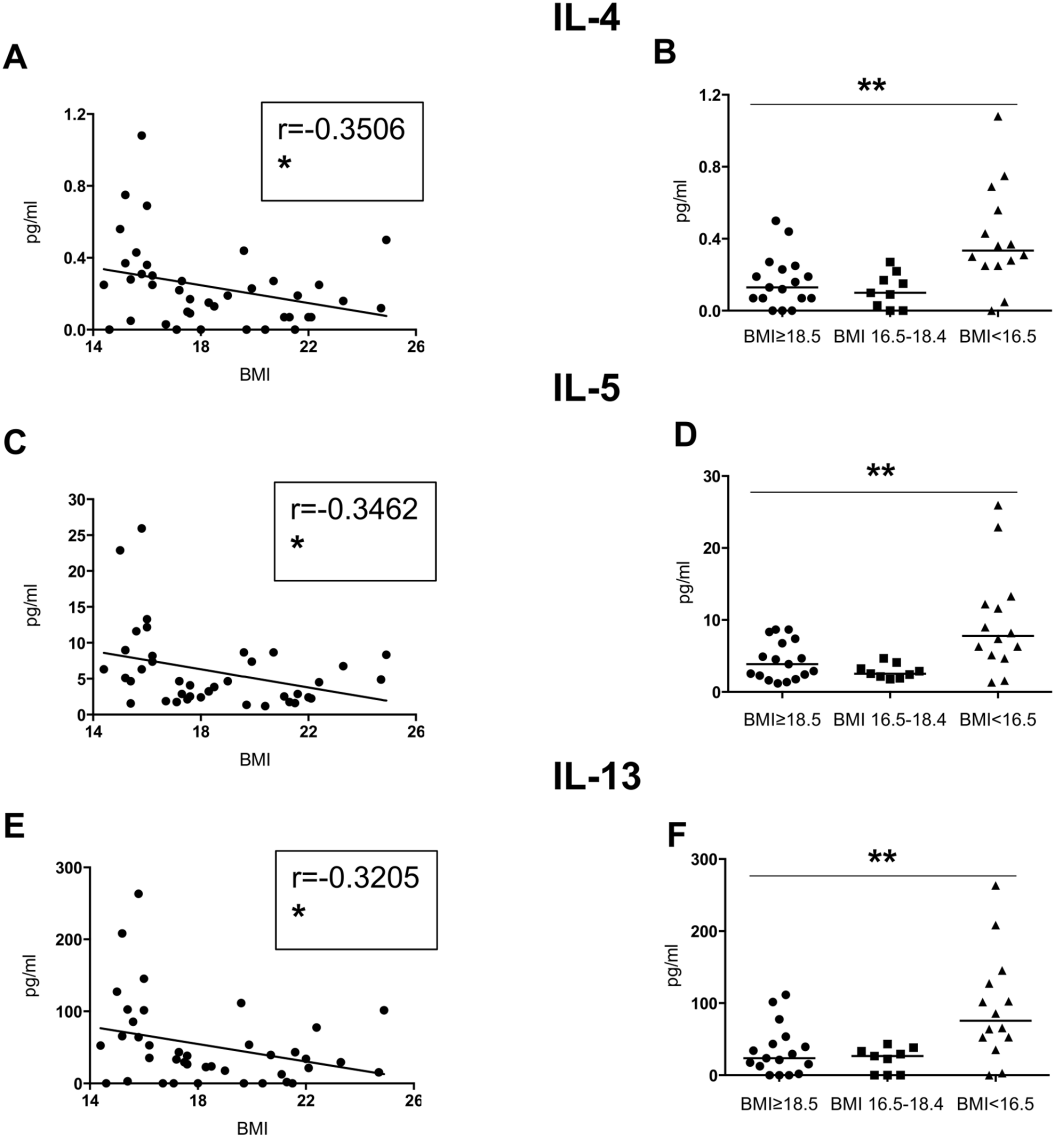
Levels of Th2 cytokines in plasma. The plasma from individuals with normal BMI (n = 17) and low BMI (n = 23, of which 9 were moderately malnourished and 14 were severely malnourished) was tested by Luminex for the levels of IL-4, IL-5 and IL-13. Fig 2A, C and E show correlations between BMI and the different cytokines and statistical significance was established using a Spearman rank test. Fig B, D and F show cytokine levels between the individuals with a normal BMI, moderately malnourished and severely malnourished individuals and statistical significance was established using a Kruskal-Wallis test.

Th2 cytokines (Table 3, Fig 2): similarly, significant negative correlations were observed between BMI and the plasma levels of IL-4, IL-5 and IL-13 between the 3 groups (Kruskal-Wallis and Spearman: Table 3 and Fig 2; Table D in S1 Data).

**Fig 3:**
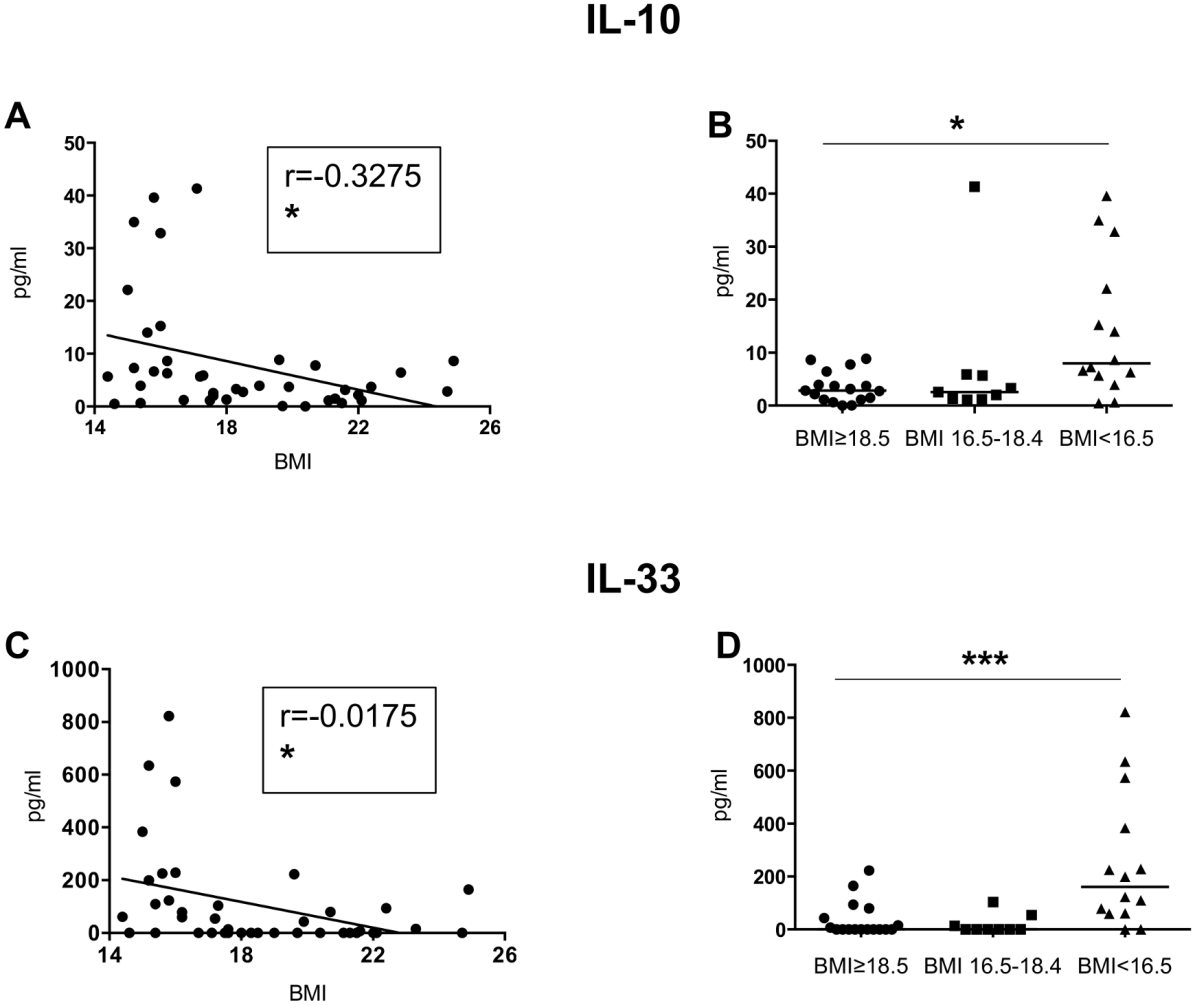
IL-10 and IL-33 levels in plasma. The plasma from individuals with normal BMI (n = 17) and low BMI (n = 23, of which 9 were moderately malnourished and 14 were severely malnourished) was tested by Luminex for the levels of IL-10 and IL-33. Fig 3A, C and E show correlations between BMI and the different cytokines and statistical significance was established using a Spearman rank test. Fig 3B, D and F show cytokine levels between the individuals with a normal BMI, moderately malnourished and severely malnourished individuals and statistical significance was established using a Kruskal-Wallis test.

IL-10 and IL-33 (Table 3, Fig 3): the levels of IL-10 and IL-33 in the plasma of the cohort followed the same trend as that of Th1 and Th2 cytokines: the correlations between BMI and IL-10 and IL-33 were significant and the levels of IL-10 and IL-33 were significantly different in-between the 3 groups (Kruskal-Wallis and Spearman: Table 3, Fig 3; Table E in S1 Data).

**Fig 4:**
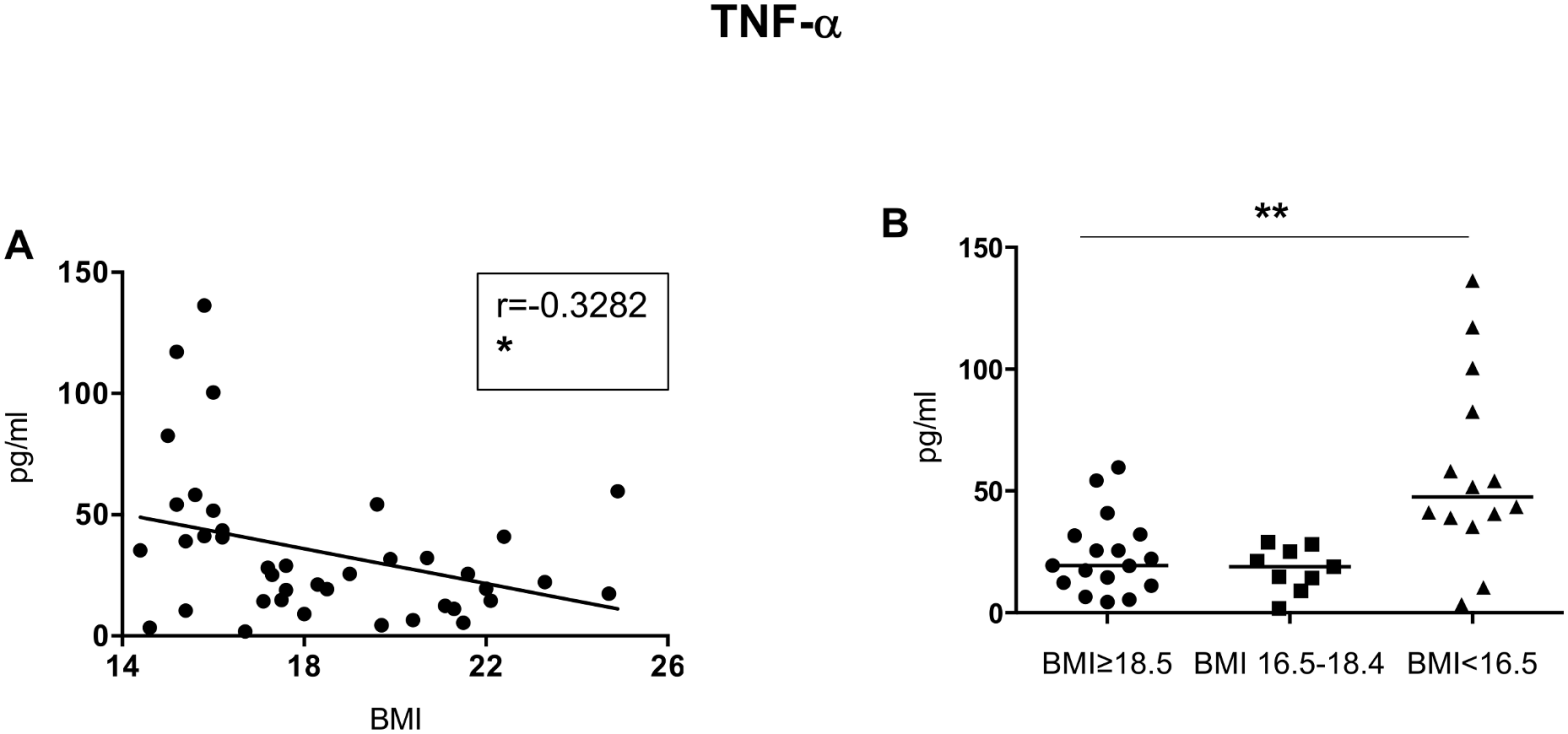
TNF-α. The plasma from individuals with normal BMI (n = 17) and low BMI (n = 23, of which 9 were moderately malnourished and 14 were severely malnourished) was tested by ELISA for the levels of IL-8. Fig 4A shows correlations between BMI and TNF-α levels and statistical significance was established using a Spearman rank test. Fig 4B shows TNF-α levels between the individuals with a normal BMI, moderately malnourished and severely malnourished individuals and statistical significance was established using a Kruskal-Wallis test.

Inflammatory mediators (Table 3, Fig 4): To determine whether inflammation was increased in malnourished individuals, we measured the plasma levels of CRP, TNF-α and IL-8. Whereas no significant differences were observed between CRP and IL-8 and BMI (Kruskal-Wallis and Spearman: Table 3; Table F in S1 Data), a clear inverse correlation was measured between TNF-α and BMI (Kruskal-Wallis and Spearman: Table 3, Fig 4; Table F in S1 Data).

These results show that malnutrition correlates with increased levels of the inflammatory mediator TNF-α.

### Arginase activity

Enhanced arginase-induced L-arginine depletion is increasingly recognized as a key mechanism of T cell suppression [33] and we have shown that increased arginase activity is a marker of disease severity in patients with visceral leishmaniasis [23] and HIV [34]. We have recently shown in an animal model of PEM that the levels of arginase activity are significantly altered in malnourished mice [35]. In human blood, arginase is expressed by neutrophils, as well as by a population of activated neutrophils; the latter is found in the PBMCs fraction following density gradient centrifugation and is therefore named low-density granulocytes (LDGs) as opposed to the normal density granulocytes (NDGs) that are found with the erythocytic fraction.

Here, we measured the levels of arginase activity by enzymatic assay in the plasma, PBMCs and neutrophils. No significant differences were observed in the levels of arginase in the plasma, PBMCs and neutrophils of all groups (Kruskal-Wallis: Table 4 and Table G in S1 Data). Since we had previously shown that CD3ζ, one of the main signalling chain of the T cell receptor, is downregulated in T cells rendered hyporesponsive following arginase-induced L-arginine depletion, we measured the expression levels of CD3ζ in CD4^+^ and CD8^+^ T cells from our cohort by flow cytometry. In line with the unchanged levels of arginase activity, the expression levels of CD3ζ were similar in all groups (Kruskal-Wallis: Table 5 and Table H in S1 Data). We have previously shown that in PBMCs, the main cells expressing arginase are LDGs, they are identified as CD15^+^arginase^+^CD14^-^ cells [27, 34]; therefore, we measured the expression levels of arginase in LDGs by flow cytometry. Our results show that the expression levels of intracellular arginase in LDGs are similar in all groups; in line with these results, the expression levels of CD63, a molecule that is upregulated during activation of neutrophils [36] and release of arginase, are similar on the LDGs from all groups (Kruskal-Wallis: Table 6 and Table H in S1 Data).

**Table 4:** Arginase activity in plasma, PBMCs and neutrophils of individuals with normal and low BMI.

BMI	Plasma	PBMCs	Neutrophils
**≥18.5**	0.0±2.2	29.0±7.9	1308±380
**16.5–18.4**	0.0±1.4	56.9±12.4	590±252
**<16.5**	0.0±1.0	39.4±12.0	781±404
***p* values (Kruskal Wallis)**	0.5968	0.9333	0.2447
**Correlation (BMI vs arginase activity) *p* values (spearman)**	0.8010	0.5599	0.1977

The plasma, PBMCs and NDGs from individuals with normal BMI (n = 17) and low BMI (n = 24, of which 10 were moderately malnourished and 14 were severely malnourished) were isolated and the activity of arginase was determined by enzymatic assay. Correlations between BMI and arginase activities were assessed using a Spearman rank test. Statistical differences in arginase activities between individuals with a normal BMI, moderately malnourished and severely malnourished individuals were established using a Kruskal-Wallis test.

**Table 5:** CD3ζ expression in CD4^+^ and CD8^+^ T cells in individuals with normal and low BMI.

BMI	CD4^+^ T cells	CD8^+^ T cells
**≥18.5**	11.1±1.2	11.3±1.2
**16.5–18.4**	12.1±2.5	9.4±2.0
**<16.5**	14.5±1.8	8.6±1.6
***p* values (Kruskal Wallis)**	0.4466	0.5636
**Correlation (BMI vs CD3**ζ**) *p* values (spearman)**	0.3675	0.5009

PBMCs from individuals with normal BMI (n = 12) and low BMI (n = 21, of which 8 were moderately malnourished and 13 were severely malnourished) were isolated and the expression levels of CD3ζ in CD4^+^ and CD8^+^ T cells were determined by flow cytometry. Correlations between BMI and CD3ζ iMFI in CD4^+^ and CD8^+^ T cells were assessed using a Spearman rank test. Statistical differences in CD3ζ MFI in CD4^+^ and CD8^+^ T cells between individuals with a normal BMI, moderately malnourished and severely malnourished individuals were established using a Kruskal-Wallis test.

**Table 6:** CD63 and arginase MFI in LDGs and NDGs in individuals with normal and low BMI.

BMI	LDGs CD63 (MFI)	NDGs CD63 (MFI)	LDGs arginase (MFI)	NDGs arginase (MFI)
**≥18.5**	9.5±0.8	8.7±0.4	91.4±4.5	290.2±30.8
**16.5–18.4**	7.8±0.7	7.9±0.7	85±10.2	261.2±51.6
**<16.5**	8.7±0.7	7.8±0.5	76.3±11.9	323.5±39.7
***p* values (Kruskal Wallis)**	0.7558	0.1917	0.8903	0.6843
**Correlation (BMI vs MFI) *p* values (spearman)**	0.7254	0.8220	0.5835	0.5378

PBMCs and NDGs from individuals with normal BMI (n = 17) and low BMI (n = 22, of which 9 were moderately malnourished and 13 were severely malnourished) were isolated and the expression levels of CD63 and arginase in LDGs and NDGs were determined by flow cytometry. Correlations between BMI and CD63 and arginase MFI in in LDGs and NDGs were assessed using a Spearman rank test. Statistical differences in CD63 and arginase MFI in LDGs and NDGs between individuals with a normal BMI, moderately malnourished and severely malnourished individuals were established using a Kruskal-Wallis test.

**Fig 5:**
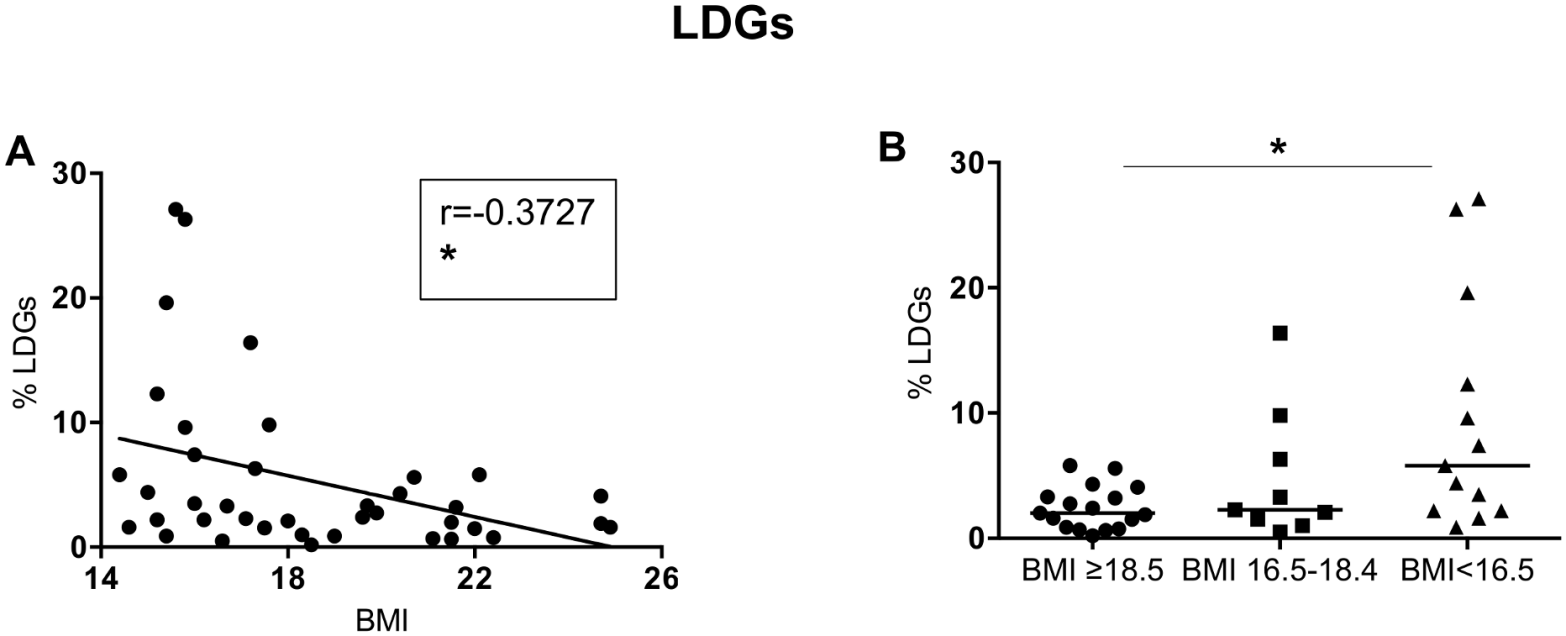
Frequency of LDGs in PBMCs. PBMCs from individuals with normal BMI (n = 17) and low BMI (n = 23, of which 9 were moderately malnourished and 14 were severely malnourished) were isolated by density gradient centrifugation and the frequency of LDGs (CD15+arginase+ cells) was determined by flow cytometry. Fig 5A shows the correlation between BMI and the frequency of LDGs and statistical significance was established using a Spearman rank test. Fig 5B shows the frequency of LDGs between the individuals with a normal BMI, moderately malnourished and severely malnourished individuals and statistical significance was established using a Kruskal-Wallis test.

Next, we measured the frequency of LDGs in the PBMCs isolated from our cohort and our results show that there is a significant negative correlation between BMI and the percentages of LDGs; the frequency was also significantly different between the 3 groups (Kruskal-Wallis and Spearman: Table 7, Fig 5; Table I in S1 Data).

**Table 7:** Percentages of LDGs in individuals with normal and low BMI.

BMI	% LDGs
**≥18.5**	2.0±0.4
**16.5–18.4**	2.3±1.8
**<16.5**	5.8±2.6
***p* values (Kruskal Wallis)**	0.0265
**Correlation (BMI vs % LDGs) *p* values (spearman)**	0.0194

PBMCs from individuals with normal BMI (n = 17) and low BMI (n = 22, of which 9 were moderately malnourished and 13 were severely malnourished) were isolated by density gradient centrifugation and the frequency of LDGs (CD15+arginase+ cells) were determined by flowcytometry. Correlation between BMI and the percentage of LDGs was assessed using a Spearman rank test. Statistical differences in arginase activities between individuals with a normal BMI, moderately malnourished and severely malnourished individuals was established using a Kruskal-Wallis test.

Finally, we measured the levels of arginase activity by enzymatic assay as well as the expression levels of CD63 and arginase by flow cytometry in LDGs. As shown in Tables 4 and 6, no significant differences were observed in all the parameters measured.

These results show that arginase activity in plasma, PBMCs and NDGs, as well as the levels of degranulation markers on NDGs and LDGs we assessed are similar on all groups; however, malnourished individuals have significantly more LDGs in their PBMCs.

### Neutrophil effector functions

**Fig 6:**
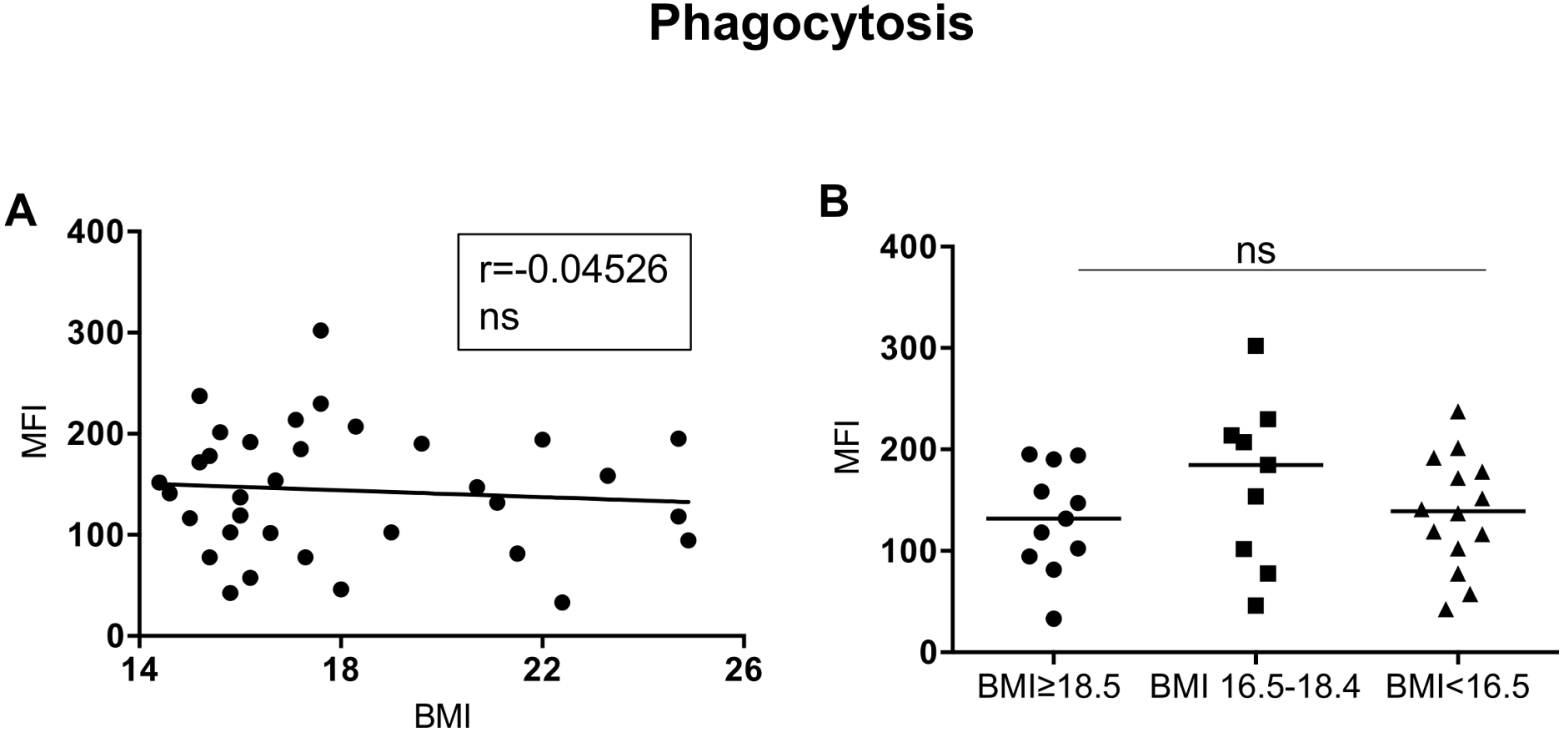
Phagocytosis. NDGs from individuals with normal BMI (n = 17) and low BMI (n = 23, of which 9 were moderately malnourished and 14 were severely malnourished) were isolated by double density gradient centrifugation NDGs and the capacity of NDGs to phagocytose particles was assessed by flow cytometry. Fig 6A shows correlations between BMI and phagocytosis and statistical significance was established using a Spearman rank test. Fig 6B shows phagocytosis between the individuals with a normal BMI, moderately malnourished and severely malnourished individuals and statistical significance was established using a Kruskal-Wallis test.

Neutrophils play a key role in the fight against invading microorganisms, via several mechanisms such as phagocytosis and production of cytotoxic molecules. We first assessed the capacity of neutrophils isolated by double Ficoll gradient to phagocytose particles. As shown in Fig 6, there is no significant correlation between the capacity of neutrophils to phagocytose and the BMI and the capacity to take up particles was similar in all groups (Kruskal-Wallis and Spearman: Table 8, Fig 6).

**Table 8:** NDGs' effector functions.

Phagocytosis	Increase MFI	ROS production	Increase MFI (Pyocyanin)	Increase MFI (LPS)
BMI		BMI		
**≥18.5**	131.7±15.7	**≥18.5**	5.0±3.3	7.1±4.7
**16.5–18.5**	184.8±27.2	**16.5–18.5**	2.8±1.3	4.2±1.7
**<16.6**	139.2±15.0	**<16.6**	1.3±0.7	1.6±0.7
***p* values (Kruskal Wallis)**	0.4696	***p* values (Kruskal Wallis)**	0.0741	0.0271
***Correlation (BMI vs increase MFI) p values (spearman)***	0.7994	***Correlation (BMI vs increase MFI) p values (spearman)***	0.0231	0.0033

NDGs were isolated by double density gradient centrifugation NDGs. The capacity of NDGs to phagocytose particles (individuals with normal BMI; n = 10 and low BMI:n = 23, of which 9 were moderately malnourished and 14 were severely malnourished) and to produce ROS (individuals with normal BMI: n = 17 and low BMI: n = 24, of which 10 were moderately malnourished and 14 were severely malnourished) were assessed by flow cytometry. Correlations between BMI and phagocytosis and BMI and ROS were assessed using a Spearman rank test. Statistical differences in phagocytosis activities and ROS production between individuals with a normal BMI, moderately malnourished and severely malnourished individuals were established using a Kruskal-Wallis test.

**Fig 7:**
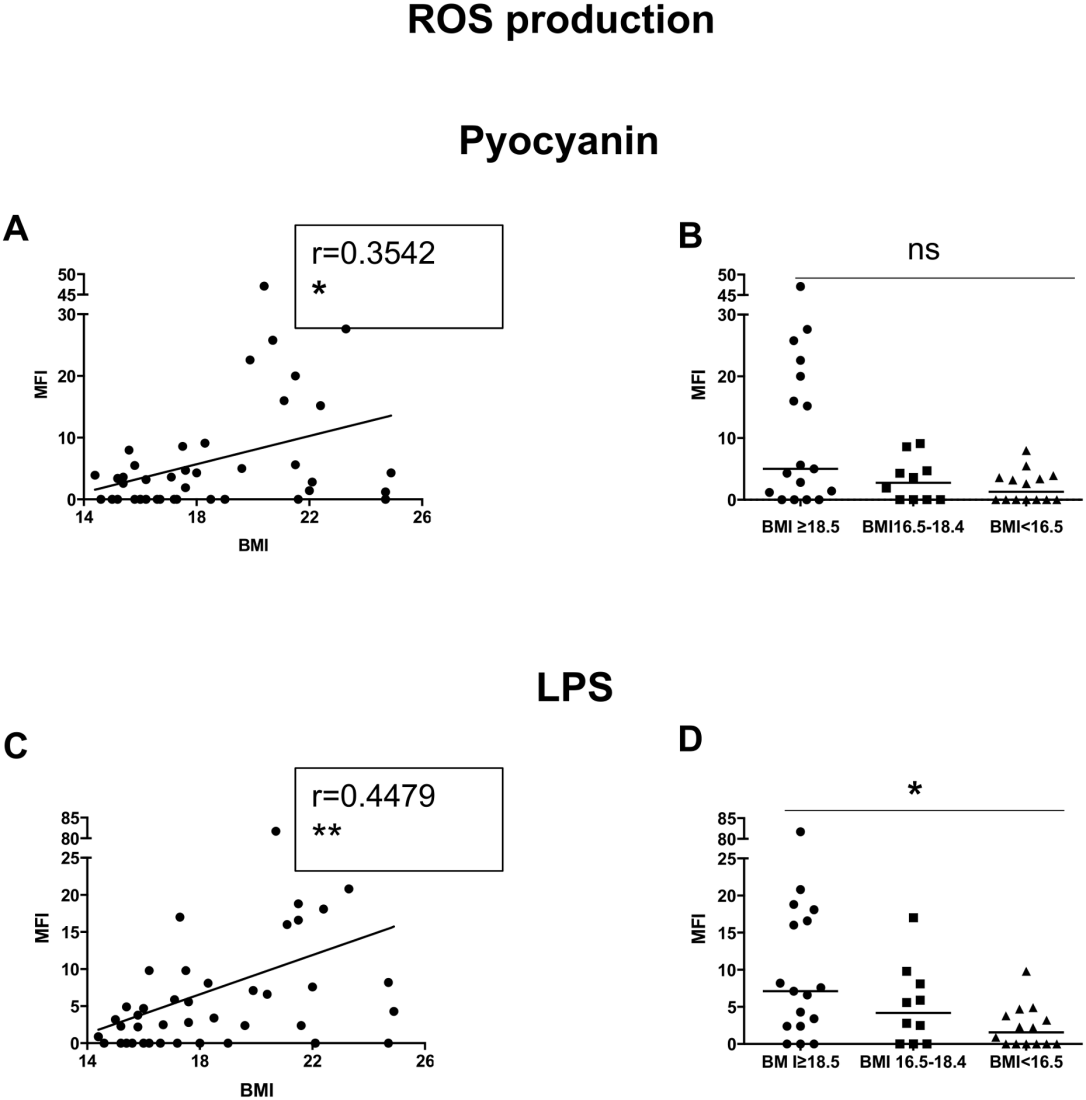
ROS production. NDGs from individuals with normal BMI (n = 17) and low BMI (n = 23, of which 9 were moderately malnourished and 14 were severely malnourished) were isolated by double density gradient centrifugation NDGs and the capacity of NDGs to produce ROS was assessed by flow cytometry. Fig 7A and C show correlations between BMI and ROS production and statistical significance was established using a Spearman rank test. Fig 7B and D show ROS production between the individuals with a normal BMI, moderately malnourished and severely malnourished individuals and statistical significance was established using a Kruskal-Wallis test.

Next we assessed the capacity of neutrophils to produce ROS in response to different stimuli: our results showed a positive correlation between the capacity of activated neutrophils to produce ROS in response to pyocyanin (Fig 7A) and LPS (Fig 7C) and BMI; furthermore, neutrophils from the malnourished group have a significantly impaired capacity to produce ROS in response to LPS (Kruskal-Wallis and Spearman: Table 8, Fig 7; Table J in S1 Data).

These results show that neutrophils from malnourished individuals display a decreased capacity to produce ROS.

## Discussion

The results of our study show that several immunological parameters are altered in "apparently healthy" malnourished individuals: we observed significantly increased production of mixed cytokines as well as impaired neutrophil effector functions. In contrast, the haematological data were similar amongst all groups, suggesting that the malnourished individuals did not have an infection, were not anaemic, neutropenic, lymphopenic or thrombocytopenic. Furthermore, the frequency and percentage of CD4^+^ and CD8^+^ T cells were similar in the malnourished healthy individuals and those with normal BMI. Little is known about haematological profiles in malnourished individuals, the main body of the work done in this area was performed in children and some studies have shown that anaemia [37, 38] can be associated with malnutrition. In addition, studies of the haematological data from patients with anorexia nervosa have also shown that anaemia and mild neutropenia are found in some patients [39, 40]. Of note, patients with anorexia nervosa included in these studies had a BMI considerably lower (e.g. 11.6–14.0) then the malnourished individuals from our study (16.1) and this might explain the differences in results.

The levels of cytokines in the plasma of malnourished individuals were significantly increased, even more so in the severely malnourished group. No switch to a preferential Th1, Th2 or inflammatory response was observed, but rather the levels of a heterogeneous spectrum of cytokines were enhanced. Inflammation has been associated to malnutrition [41, 42], and consistent with this association, our results show increased levels of TNF-α in the plasma. It is possible that anti-inflammatory cytokines, such as IL-10 are produced to regulate inflammation and therefore prevent a damaging systemic inflammation. Similarly, production of Th1 cytokines such as IFN-γ might be increased to down-modulate Th2 cytokines and vice and versa. IL-33, a pleiotropic cytokine of the IL-1 family that was originally considered to be an inducer of Th2 responses has also been associated with the repression of Th1 immune response, tissue homeostasis and inflammation (reviewed in [43]). In the plasma of the severely malnourished individuals, IL-33 was significantly increased. Therefore, in the case of a malnourished patient suffering from an infectious disease such as visceral leishmaniasis, the increased IL-33 might impact on Th1 immune responses, as well as contribute to the inflammation. In contrast to the elevated levels inflammatory mediators such as TNF-αand IL-33, the levels of CRP were not significantly altered: and indeed, acute phase proteins are not consistently associated with weight loss and calorie restriction [41]; it could also be due to the fact that the malnourished individuals participating in our study were "apparently healthy" and that their BMI (median: 16.1) was not excessively low.

Autophagy is a highly conserved cellular process that plays a crucial role in many biological events to adapt to a changing environment such as nutrient starvation: it is responsible for the degradation of organelles within lysosomes and cytosolic proteins, that will be recycled to provide nutrients and energy to promote survival [44]. However, with prolonged nutrient starvation, cells will eventually die [45]. For example, in patients with anorexia nervosa, autophagy appears to be initially beneficial by allowing the cells to manage the nutrient deficiency; however, on the long term, acute liver insufficiency with features of autophagic cell death can be observed [46]. Cyokines play an important role in the promotion or the inhibition of autophagy, with Th1 cytokines acting as promoters and Th2 and anti-inflammatory cytokines acting as inhibitors. In addition, autophagy can also promote or inhibit the release of cytokines (reviewed in [47]). Therefore it is likely that the increased levels of cytokines we observe in the plasma of severely malnourished individuals result from an uncontrolled balance between the protection of nutrient starved cells by autophagy, but also the detrimental effects of autophagy due to prolonged starvation. This inability to control the increased production of cytokines might cause collateral damages to the surrounding cells and tissues; and, when a malnourished individual get infected with a pathogen, the immune response associated with the attempted clearance of the pathogen will synergize with that associated with malnutrition. This is likely to result in an excessive inflammatory response, similar to that associated to severe leishmaniasis [24] and other infectious diseases such as malaria [48].

Our results in an experimental model of protein energy malnutrition have shown that arginase, an enzyme with immunomodulatory properties, is increased in the monocytes from malnourished mice [35]. In humans arginase is constitutively expressed by neutrophils, but not by monocytes [33] and we did not find increased levels of arginase in plasma or lower levels in PBMCs or neutrophils, which would have indicated that arginase had been released in the microenvironment. Arginase is localized in azurophilic granules in neutrophils [33], which express CD63; following activation neutrophils degranulate and the release of the arginase-containing azurophilic granules results in the incorporation of CD63 into the cell surface membrane of neutrophils [36]. The degree of neutrophil activation and degranulation depend on the strength of the activating signal: the order of granule release follows a strict hierarchy requiring increasing activation: 1) secretory granules; 2) gelatinous (tertiary) granules; 3) specific (secondary) granules and 4) azurophilic (primary) granules. Our results suggest therefore that the neutrophils from malnourished individuals have not been fully activated, as the expression levels of CD63 expression were not increased and intra-cellular arginase levels were not decreased. However, we did find a significant correlation between BMI and LDGs and an increased frequency of LDGs in the PBMCs of malnourished individuals. These cells have been previously shown to interfere with T cell activation via mechanisms such as arginase-induced L-arginine depletion [33, 49] or upregulation of PD-L1 [50]. Therefore an increased frequency of cells with immunosuppressive properties will be an obvious disadvantage in a malnourished individual acquiring an infectious disease.

Neutrophils are highly versatile cells, which play a crucial role in the induction and the resolution of inflammation, the regulation of immune response, and the elimination of pathogen by mechanisms such as phagocytosis and production of toxic molecules. However, their role in human adult malnutrition is poorly characterized. Here, we show that the ability of neutrophils from malnourished individuals to produce ROS was significantly impaired, suggesting that neutrophils from malnourished individuals are less capable to kill pathogens. This will have obvious harmful consequences on the capacity of neutrophils from a malnourished individual to control the growth of pathogenic microorganisms.

Taken together, our results reveal that malnutrition in "apparently healthy" individuals impairs innate immune functions as well as the cytokine and inflammatory environment, and might therefore contribute to increased incidence of infectious diseases and/or increased disease severity. These results are relevant for the study of disease susceptibility and severity during infectious diseases; particularly in our field of interest, non-healing visceral leishmaniasis, that is characterized by high levels of cytokine production, acute inflammatory response, T cell hyporesponsiveness and dysfunctional neutrophils. In the North West of Ethiopia, where our studies are taking place, VL patients treated in the Leishmaniasis Treatment and Research Center are from a socio-demographic background that is strongly associated with poverty and malnutrition. It is therefore of paramount importance to obtain a better understanding of the impact of malnutrition-induced increased disease incidence and/or severity, as well as disease-induced malnutrition on immune responses, as to design dietary interventions that might improve immune responses and resistance to infectious diseases in malnourished individuals.

## Supporting Information

S1 Data Tables A-J. (DOCX)
