# Supplementary material for: Malnutrition in Healthy Individuals Results in Increased Mixed Cytokine Profiles, Altered Neutrophil Subsets and Function
Source: PLoS One. 2016 Aug 22;11(8):e0157919. doi: 10.1371/journal.pone.0157919 (PMC4993519; doi:10.1371/journal.pone.0157919)
Supplement: S1 Data — (DOCX) [file pone.0157919.s001.docx]

**Supplementary data**

**Table A**: Multivariate Analysis of Variance (MANOVA): Haematological profile of individuals with a normal BMI, moderately or severely malnourished individuals.

| **Source** | **Dependent variable** | **df** | **SS** | **MS** | **F value** | **p-value** |
| --- | --- | --- | --- | --- | --- | --- |
| **BMI** | **WBC** | 2 | .693 | .346 | .083 | .921 |
|  | **Hgb** | 2 | .656 | .328 | .117 | .890 |
|  | **Platelets** | 2 | 2222.210 | 1111.105 | .348 | .709 |
|  | **Neutrophils** | 2 | 1.076 | .538 | .234 | .793 |
|  | **Lymphocytes** | 2 | .108 | .054 | .161 | .852 |
|  | **RBC** | 2 | .089 | .045 | .143 | .867 |
| **Error** | WBC | 29 | 121.087 | 4.175 |  |  |
|  | **Hgb** | 29 | 81.210 | 2.800 |  |  |
|  | **Platelets** | 29 | 92689.790 | 3196.200 |  |  |
|  | **Neutrophils** | 29 | 66.778 | 2.303 |  |  |
|  | **Lymphocytes** | 29 | 9.786 | .337 |  |  |
|  | **RBC** | 29 | 9.044 | .312 |  |  |

**Table B**: Multivariate Analysis of Variance (MANOVA): percentages and ratio of CD4^+^ and CD8^+^ T cells in individuals with a normal BMI, moderately or severely malnourished individuals.

| **Source** | **Dependent variable** | **df** | **SS** | **MS** | **F value** | **p-value** |
| --- | --- | --- | --- | --- | --- | --- |
| **BMI** | **%CD4^+^ T cells** | 2 | 102.331 | 51.166 | .772 | .471 |
|  | **%CD8^+^ T cells** | 2 | 247.786 | 123.893 | 2.046 | .147 |
| **Error** | **%CD4^+^ T cells** | 30 | 1989.338 | 66.311 |  |  |
|  | **%CD8^+^ T cells** | 30 | 1816.260 | 60.542 |  |  |

**Table C:** Multivariate Analysis of Variance (MANOVA): Th1 cytokines in the plasma of individuals with a normal BMI, moderately or severely malnourished individuals.

| **Source** | **Dependent variable** | **df** | **SS** | **MS** | **F value** | **p-value** |
| --- | --- | --- | --- | --- | --- | --- |
| **BMI** | **IFN-**γ | 2 | 37307.312 | 18653.656 | 9.351 | **.001** |
|  | **IL-12** | 2 | 31757.633 | 15878.817 | 7.198 | **.002** |
|  | **IL-2** | 2 | 920.313 | 460.157 | 8.397 | **.001** |
| **Error** | **IFN-**γ | 37 | 73809.687 | 1994.856 |  |  |
|  | **IL-12** | 37 | 81622.099 | 2206.003 |  |  |
|  | **IL-2** | 37 | 2027.577 | 54.799 |  |  |

**Table D:** Multivariate Analysis of Variance (MANOVA): Th2 cytokines in the plasma of individuals with a normal BMI, moderately or severely malnourished individuals.

| **Source** | **Dependent variable** | **df** | **SS** | **MS** | **F value** | **p-value** |
| --- | --- | --- | --- | --- | --- | --- |
| **BMI** | **IL-4** | 2 | .633 | .211 | 5.208 | **.004** |
|  | **IL-5** | 2 | 336.768 | 112.256 | 5.131 | **.005** |
|  | **IL-13** | 2 | 38063.394 | 12687.798 | 4.949 | **.006** |
| **Error** | **IL-4** | 36 | 1.459 | .041 |  |  |
|  | **IL-5** | 36 | 787.641 | 21.879 |  |  |
|  | **IL-13** | 36 | 92284.673 | 2563.463 |  |  |

**Table E**: Multivariate Analysis of Variance (MANOVA): IL-10 and IL33 in the plasma of individuals with a normal BMI, moderately or severely malnourished individuals.

| **Source** | **Dependent variable** | **df** | **SS** | **MS** | **F value** | **p-value** |
| --- | --- | --- | --- | --- | --- | --- |
| **BMI** | **IL-10** | 2 | 894.605 | 447.303 | 4.462 | **.018** |
|  | **IL-33** | 2 | 439850.419 | 219925.210 | 8.616 | **.001** |
| **Error** | **IL-10** | 37 | 3708.832 | 100.239 |  |  |
|  | **IL-33** | 37 | 944472.598 | 25526.286 |  |  |

**Table F:** Multivariate Analysis of Variance (MANOVA): Inflammatory mediators in the plasma of individuals with a normal BMI, moderately or severely malnourished individuals.

| **Source** | **Dependent variable** | **df** | **SS** | **MS** | **F value** | **p-value** |
| --- | --- | --- | --- | --- | --- | --- |
| **BMI** | **TNF-α** | 2 | 14489.928 | 7244.964 | 12.695 | **.000** |
|  | **IL-8** | 2 | 18.921 | 9.460 | 1.220 | .307 |
|  | **CRP** | 2 | 18646521.683 | 9323260.841 | .059 | .943 |
| **Error** | **TNF-α** | 36 | 20544.635 | 570.684 |  |  |
|  | **IL-8** | 36 | 279.148 | 7.754 |  |  |
|  | **CRP** | 36 | 5722353612.16 | 158954267.01 |  |  |

**Table G**: Multivariate Analysis of Variance (MANOVA): Arginase activity in plasma, PBMCs and neutrophils of individuals with a normal BMI, moderately or severely malnourished individuals.

| **Source** | **Dependent variable** | **df** | **SS** | **MS** | **F value** | **p-value** |
| --- | --- | --- | --- | --- | --- | --- |
| **BMI** | **Plasma** | 2 | 11.166 | 5.583 | .305 | .739 |
|  | **PBMCs** | 2 | 317.641 | 158.820 | .162 | .851 |
|  | **Neutrophils** | 2 | 8750.404 | 4375.202 | .226 | .799 |
| **Error** | **Plasma** | 33 | 604.831 | 18.328 |  |  |
|  | **PBMCs** | 33 | 32342.596 | 980.079 |  |  |
|  | **Neutrophils** | 33 | 639459.755 | 19377.568 |  |  |

**Table H:** Multivariate Analysis of Variance (MANOVA): CD3ζ expression in CD4^+^ and CD8^+^ T cells in individuals with a normal BMI, moderately or severely malnourished individuals.

| **Source** | **Dependent variable** | **df** | **SS** | **MS** | **F value** | **p-value** |
| --- | --- | --- | --- | --- | --- | --- |
| **BMI** | **CD4^+^ T cells** | 2 | 71.484 | 35.742 | 1.048 | .363 |
|  | **CD8^+^ T cells** | 2 | 40.903 | 20.452 | .807 | .456 |
| **Error** | **CD4^+^ T cells** | 30 | 1023.397 | 34.113 |  |  |
|  | **CD8^+^ T cells** | 30 | 760.072 | 25.336 |  |  |

**Table I**: Multivariate Analysis of Variance (MANOVA) for CD63 and arginase MFI in LDGs and NDGs in individuals with a normal BMI, moderately or severely malnourished individuals.

| **Source** | **Dependent variable** | **df** | **SS** | **MS** | **F value** | **p-value** |
| --- | --- | --- | --- | --- | --- | --- |
| **BMI** | **LDGs CD63 MFI** | 2 | 8.515 | 4.258 | .601 | .554 |
|  | **LDGs arginase MFI** | 2 | 167.493 | 83.747 | .093 | .911 |
|  | **NDGs CD63 MFI** | 2 | 7.026 | 3.513 | 1.093 | .346 |
|  | **NDGs arginase MFI** | 2 | 10830.451 | 5415.225 | .289 | .751 |
| **Error** | **LDGs CD63 MFI** | 38 | 269.374 | 7.089 |  |  |
|  | **LDGs arginase MFI** | 38 | 34212.894 | 900.339 |  |  |
|  | **NDGs CD63 MFI** | 35 | 112.456 | 3.213 |  |  |
|  | **NDGs arginase MFI** | 35 | 656054.692 | 18744.420 |  |  |

**Table J**: Multivariate Analysis of Variance (MANOVA) for ROS production by neutrophils from individuals with a normal BMI, moderately or severely malnourished individuals.

| **Source** | **Dependent variable** | **df** | **SS** | **MS** | **F value** | **p-value** |
| --- | --- | --- | --- | --- | --- | --- |
| **BMI** | **Increase in MFI**  **(pyocyanin)** | 2 | 590.589 | 295.295 | 3.707 | **.034** |
|  | **Increase in MFI**  **(LPS)** | 2 | 275.929 | 137.964 | 4.754 | **.015** |
| **Error** | **Increase in MFI**  **(pyocyanin** | 36 | 2867.770 | 79.660 |  |  |
|  | **Increase in MFI**  **(LPS)** | 36 | 1044.748 | 29.021 |  |  |
